# Primary alveolar rhabdomyosarcoma of the bone: two cases and review of the literature

**DOI:** 10.1186/s13000-016-0552-9

**Published:** 2016-10-18

**Authors:** Petra Balogh, Rita Bánusz, Monika Csóka, Zsófia Váradi, Edit Varga, Zoltán Sápi

**Affiliations:** 1 1st Department of Pathology and Experimental Cancer Research, Semmelweis University, Üllői út 26, Budapest, H-1085 Hungary; 2 2nd Department of Pediatrics, Semmelweis University, Tűzoltó utca 7-9, Budapest, H-1094 Hungary; 3 Magnetic Resonance Research Center, Semmelweis University, Tűzoltó utca 7-9, Budapest, H-1094 Hungary

**Keywords:** Alveolar rhabdomyosarcoma of bone, FOXO-1, Clinico-pathological entity

## Abstract

**Background:**

Rhabdomyosarcoma (RMS) is a malignant tumor of mesenchymal origin and comprises the largest category of soft-tissue sarcomas both in children and adolescents. From a pediatric oncology point of view, RMS has traditionally been classified into alveolar (ARMS) and embryonal (ERMS) subtypes. The anatomical localization of the tumor may vary, but commonly involve the head/neck regions, male and female urogenital tract or the trunk and extremities.

**Case presentation:**

Here, we report two challenging cases involving 17- and 9-years-olds males where diffuse and multiplex bone lesions suggested either a hematological disease or a primary bone tumor (mesenchymal chondrosarcoma). Biopsies, proved a massive infiltration of the bone marrow cavity with rhabdomyosarcoma. In both cases, the ARMS subtype was confirmed using FOXO1 break-apart probes (FISH). Radiological examination could not identify primary soft tissue component in any localization at the time of diagnosis in either cases.

**Conclusions:**

Primary alveolar rhabdomyosarcoma of the bone as a subtype of ARMS, seems to be a distinct clinico-pathological entity with challenging diagnostic difficulties and different, yet better, biological behavior in comparison to soft tissue ARMS. However, it is difficult to be characterized or predict its prognosis and long-term survival as only sporadic cases (four) were reported so far.

## Background

Rhabdomyosarcoma (RMS) is among the most common soft tissue sarcomas in childhood and adolescence with 4.5 new cases/1 million person/year in the USA and incidences in Europe share similar numbers [1, 2]. It is a high-grade malignancy that primarily involves the head and neck region, the urogenital tract or may develop in soft tissues of the trunk or extremities. Histologically, RMS is comprised of four subtypes; among which embryonal and alveolar RMSs are the most common ones under the age of 20, while pleomorphic and spindle cell variants of the tumor may also occur in adults, with a peak at the 4th-5th and 6th -7th decades of lifetime, respectively. RMS is a high-grade malignancy and the subtype determines the prognosis of the disease. While embryonal RMS has a better outcome (5-year survival rate of 82 %), the alveolar variant of the tumor has a worse prognosis (5-year survival rate of 65 %) which is presumably associated with the cytogenetic aberrations this latter subtype carry [3, 4]. Alveolar RMS can be characterized by a recurrent cytogenetic alteration involving FOXO-1 and PAX3 or PAX7 genes, and the consecutive translocations (t(2;13) or t(1;13) respectively) lead to the excess synthesis of fusion proteins with oncogenic effects [5, 6].

Available data about primary bone ARMS is limited due to the fact that so far only four cases were found in literature reporting fusion-positive alveolar RMS confined to the bone marrow [7–10]. Thus, it is difficult to predict the disease course, the biological behavior and its characteristics. Nonetheless, according to these reports as well as our experiences, primary bone ARMS seems to have a better prognosis and survival rate compared to its soft tissue counterpart. Here we report two further cases of primary ARMS of the bone that posed a diagnostic challenge both from a clinical as well as a pathological point of view.

## Case presentations 

### Clinical findings of Case 1

**Fig. 1:**
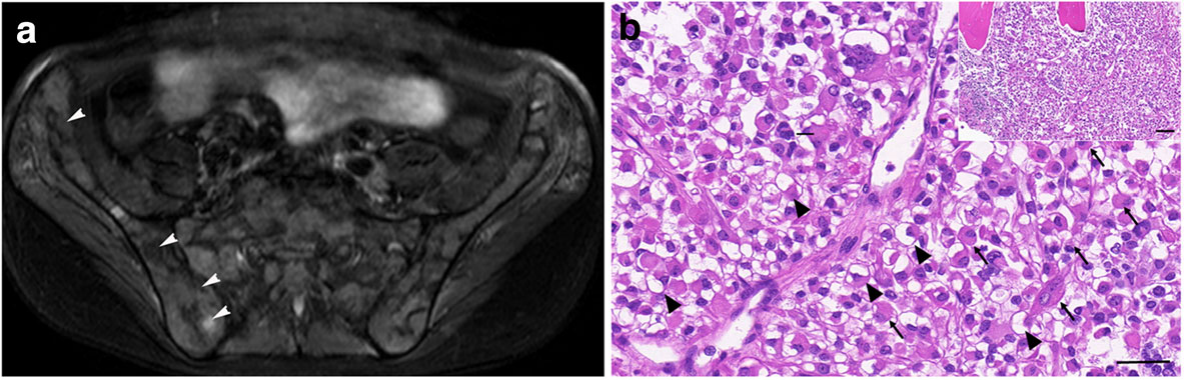
Radiological image and hematoxylin-eosin stained specimen of the tumor. **a** Axial T2 SPAIR image of pelvis shows diffuse patchy infiltration (*arrowheads*) of the bone marrow. Some small necrotic areas are also visible. **b** The HE stained biopsy sample shows highly cellular infiltrate among the bony trabeculae repelling the normal hematopoietic cells (insert image). With higher magnification, the monomorphic tumor cells have a characteristic eosinophilic cytoplasm, however tumor cells also show intracytoplasmic vacuolization (*arrowheads*). The nuclei of tumor cells are eccentric in position, but rather identical in size and own a finely granulated, basophilic nuclear structure (*arrows*). Note that the organization of tumor cells represent a somewhat nest-like pattern, but lack fine fibrovascular stroma, that is characteristic of the solid variant of alveolar RMS. Bars indicate 50 μm, insert 100 μm

A 17-year-old male with Crohn’s disease in his medical history, presented with fever, weight loss and lower back pain; experienced over a period of 1–2 weeks. He was found to have elevated inflammatory markers and serious hypercalcemia with impaired renal function. Bone scintigraphy, lumbar spine and pelvic MRI revealed disseminated, diffuse infiltration of the bone marrow which primarily raised the suspicion of lymphoma (Fig. 1a). Repeated bone marrow biopsies (iliac crests) confirmed ARMS (Grade III). A primary soft tissue tumor was never found. During chemotherapy according to CWS-2012 Protocol’s metastatic arm, dose reduction and modifications of cytostatic drugs, intensive care and hemodialysis were required several times due to serious arrhythmias and renal insufficiency caused by osteolysis-induced hypercalcemia. Despite the appropriate, aggressive chemotherapy, his disease showed progression that could be delayed temporarily by RANKL inhibitor denosumab monotherapy for a four month period. We lost him seven months after the initial symptoms.

### Pathological findings of Case 1

**Fig. 2:**
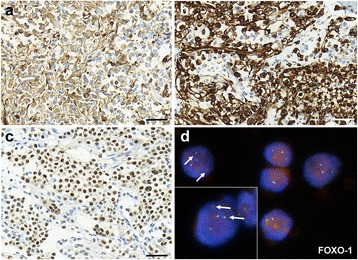
Immunohistochemical and molecular characterization of tumor cells. **a** Tumor cells show diffuse vimentin positivity, as well as diffuse and strong cytoplasmic and nuclear labelling could be observed with myogenic markers desmin and Myf-4, respectively (**b**–**c**). The result of FOXO-1 gene break-apart FISH probe demonstrates divided green and red signals indicating translocation of the affected gene (**d**). Bars indicate: 50 μm

Histological examination of the second bone marrow biopsy (iliac crests) revealed solid sheets of tumor cells infiltrating the entire bone marrow replacing and expelling the normal hematopoietic cells. The highly cellular infiltrate showed no special arrangement, although fine fibro-vascular stroma could focally be identified. The monomorphic, poorly differentiated tumor cells had round, vesicular nuclei with fine chromatin content and were localized at the periphery of the cells, in an eccentric position (Fig. 1b). The cytoplasm of most of the tumor cells possessed either an eosinophilic appearance or abundant intracytoplasmic vacuoles could be seen. While tumor cells did not show striation, the overall morphology suggested rhabdomyoblast-like differentiation (Fig. 1b). Although, by examining a HE specimen, a hematological malignancy could be ruled out, further immunohistochemical (IHC) tests were needed to characterize the phenotype of the tumor cells. The results of IHC showed diffuse vimentin positivity as well as the cells gave substantial cytoplasmic and nuclear labelling with both rhabdomyogen markers, desmin and Myf-4, respectively (Fig. 2a–c). The pan-cytokeratin and TFE-3 reactions were negative as well as INI-1 was retrained; by which alveolar soft part sarcoma or rhabdoid tumor as a differential diagnostic possibility could be ruled out. As the overall pattern of the tumor was not typical for neither embryonal nor alveolar RMS, we further performed a FOXO-1 break-apart FISH probe as the aforementioned gene is known to be involved and is consistently associated with the alveolar subtype of RMS. Indeed, we detected the translocation and break-apart signals involving FOXO-1 (Fig. 2d). Based on the histological and molecular findings as well as extended radiological examinations not proving a primary soft tissue tumor, the diagnosis of primary ARMS (solid variant) of the bone was made.

### Clinical background of Case 2

**Fig. 3:**
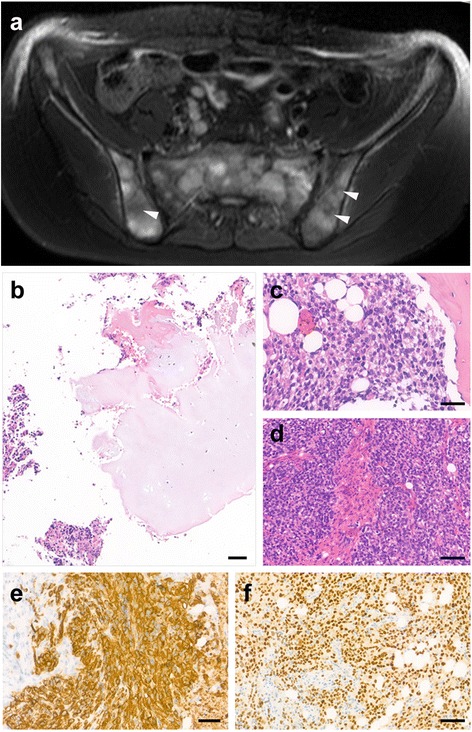
Radiological image and histopathology of Case 2. **a** On the axial T2 SPAIR image of the pelvic bone multiple, partially coalesced tumor nodules are visible in the bone marrow. Some of them (arrowheads) show central necrosis. **b** There were foci of tumor islands with atypical chondrogenic differentiation surrounded by round/spindle shape tumor cells. **c**–**d** Besides that, expansive sheet like pattern with solid nests could also be identified that were divided by fine fibrovascular septa. The infiltrate displaced the normal hematopoiesis of the bone marrow. **e**–**f** The diffuse and intensive cytoplasmic desmin and nuclear Myf-4 positivity proved rhabdomyosarcoma differentiation of tumor cells. Bars indicate: 50 μm

A 9-year-old male was admitted to the hospital presented with recurrent fever, lower back and right lower limb pain, experienced over the period of a month. He was found to have mild anemia and elevated inflammatory markers. Imaging studies revealed disseminated multiplex bone lesions involving the entire vertebral column, pelvic bones, ribs, skull, the distal part of the right femur and the proximal part of the right tibia (Fig. 3a). These findings and the lack of primary soft tissue manifestation raised the possibility of Ewing-sarcoma or malignant lymphoproliferative disease. Initially, the patient required intensive therapy for serious hypercalcemia and its complications due to osteolysis. The initial histological diagnosis was mesenchymal chondrosarcoma (Grade III), but the atypical clinical findings made histological revision necessary which, in turn, confirmed alveolar rhabdomyosarcoma with bone marrow involvement. A primary soft tissue tumor could not be identified. The therapeutic response was excellent in relation to the first-line chemotherapy given according to CWS-2009 Protocol’s metastatic arm as control MRI and PET/CT revealed complete remission. Seven months after finishing the first-line therapy, a relapse of the primary disease was confirmed, localized to the distal femur and proximal tibia on the right side. Second-line therapy was given according to CWS-2012 relapse protocol and based on the proven ALK-positivity of the tumor, ALK inhibitor crizotinib was permitted as an off-label drug for maintenance therapy for 10 months. In the fourth month of crizotinib treatment multiplex metastases were confirmed. Based on the proven increased mTOR activity of the previous biopsy specimen (iliac crests), mTOR inhibitor temsirolimus was given for 3 months. Due to disease progression, both targeted therapies were stopped and 30 months after the primary diagnosis, we lost the patient.

### Pathological findings of Case 2

The biopsy sample that was taken from the tibia showed different morphological patterns and areas that made the overall histological picture misleading: among the bony trabeculae, a cellular tumor infiltrate could be identified and the cells were arranged in solid sheets replacing the normal hematopoiesis. Other foci of the tumor showed intramedullary cartilage islands around which spindle or ovoid shape tumor cells formed a cohesive structure; allowing mesenchymal chondrosarcoma diagnosis. Besides this, however, some areas of the tumor formed solid sheets of tumor islands that were divided by fine fibro-vascular stroma (Fig. 3b–d). The cytomorphology was identical with a so called “small round blue cell tumor”. Considering that neither the age nor the dissemination of the process (multiplex bony lesions) were typical for mesenchymal chondrosarcoma, we further evaluated the phenotype of the tumor cells with several IHC tests. The cells showed cytoplasmic positivity with vimentin as well as intensive and diffuse cytoplasmic desmin and nuclear Myf-4 reactions being observed (Fig. 3e–f). To further characterize the subtype of RMS without an identifiable soft tissue component, we performed a FOXO-1 break apart FISH DNA probe that evaluated the translocation involving FOXO-1. Concerning the clinical and pathological findings, the final diagnosis of primary alveolar rhabdomyosarcoma of the bone was made.

## Discussion

Alveolar rhabdomyosarcoma is a high grade neoplasm that has the worst prognosis amongst other subtypes of RMSs (despite combined surgical and chemo/radiotherapy), especially in fusion-positive cases when FOXO-1 gene is involved. It is generally known that the overall outcomes for patients with soft tissue ARMS is worse than in patients with ERMS - even with aggressive multimodal therapy [4]. The prognostic factors defining the outcome of patients with RMS includes the following parameters: patient’s age, site of origin, tumor size, resectability, presence of metastases, number of metastatic sites or tissues involved, presence or absence of regional lymph node involvement, delivery of radiation therapy in selected cases, the unique biological characteristics of RMS tumor cells and, lastly the histological subtype. [11–14]. Regarding the histopathological subtype, there is a significant difference between the 5-year survival with ERMS (82 %) and soft tissue ARMS (65 %) [4]. Besides this, patients with (soft tissue) ARMS who have regional lymph node involvement face a worse outcome (5-year failure-free survival: 43 %) as compared to patients lacking lymph node involvement (5-year failure-free survival: 73 %) [15].

Although the previously reported four cases of primary ARMS (as well as our current two cases) show a better survival rate compared to its soft tissue counterpart, it still causes difficulties in precisely characterizing this tumor type. One reason is the low number of reported cases, while a major problem alongside this is that even data contained within medical literature is confusing with regards to ARMS classification. It distinguishes fusion-positive and fusion-negative cases; however, there is a tendency that fusion-negative cases should be considered in practical terms ERMS [16]. Until this tendency is not generalized and accepted in routine diagnostic pathology, there will be cases influencing and altering the results exhibited in statistics.

In the case of (soft tissue) alveolar RMS it is known that it commonly infiltrates the bone marrow [17], causing a diagnostic challenge (both in childhood and adult cases), as it can mimic the symptoms of either a hematological malignancy or a primary bone tumor; therefore, biopsy sampling is necessary in each and every case. The most common differential diagnostic problems (considering the localization and/or age) are as follows: Ewing sarcoma, non-Hodgkin lymphoma, mesenchymal chondrosarcoma and the small cell variant of osteosarcoma. While the morphology of tumor cells are similar (small, round cells), the pattern of infiltration or the accompanying component of the tumor (neoplastic osteoid or hyaline cartilage in small cell variant of OS and mesenchymal chondrosarcoma, respectively) as well as special cytomorphological features such as intracytoplasmic vacuoles or striation of the tumor cells (like in RMS) may sometimes suggest the differentiation lineage. Besides the careful examination of HE stained samples and morphological analysis, ancillary techniques are essential in these cases in order to give a definitive diagnosis. The combination of IHC tests including LCA, vimentin, desmin and CD99 is useful to primarily assess the phenotype of the tumor cells. Although IHC evaluation is sufficient and may lead to a final diagnosis, in most of the cases further molecular examinations such as flow cytometry (especially in hematological diseases) or genetic analysis with regard to gene fusion status (e.g. in Ewing sarcoma and ARMS) are now part of the routine diagnostic panel [18, 19].

The natural history of primary alveolar RMS of bone may show individual variations, but our current cases, together with the other four reported ones [7–10], suggest a better overall prognosis as compared to soft tissue ARMS (Table 1). Primary alveolar rhabdomyosarcoma of the bone as a subtype of ARMS seems to be a distinct clinico-pathological entity. We wish to stimulate the scientific community into publishing and following-up similar cases. With this proposal, there might be more available data to predict not only the biological behavior and prognosis of the disease, but also to develop and set up further chemotherapeutical combinations that may increase the overall survival of the patients in the future.

**Table 1:** Reported cases of primary alveolar rhabdomyosarcoma of the bone so far without identifiable soft tissue component

Case	Reference	Age	Sex	Follow-up/survival (months)	Treatment	Tumor localization
1	Yamaguchi et al. 2007 [9]	14	m	8 (s)	-Etoposide	Disseminated BM infiltration, not specified
					-Cyclophosphamide	
					-Pirarubicin	
					-Cisplatin	
					-Vincristine	
2	Jani et al. (2009) [7]	16	m	8 (s)	-VP16	Disseminated BM infiltration, not specified
					-Ifosfamide	
					-Vincristine	
					-Adriamycin	
					-Cyclophosphamide	
3	Kern et al. (2015) [10]	52	f	12 (s)	Not detailed	BM infiltration, not specified
4	Karagiannis et al. (2015) [8]	61	f	7 (f)	-Topotecan	BM infiltration, not specified
					-Cyclophosphamide	
					-Vinorelbine (Monotherapy-later)	
5	Case 1 (current report)	17	m	7 (s)	-Ifosfamid	Diffuse BM infiltration
					-Carboplatin	
					-Etoposid	
					-Vincristin	
6	Case 2 (current report)	9	m	30 (s)	-Ifosfamid	Tibia, femur, pelvic bones, vertebrae
					-Etoposid	
					-Carboplatin	
					-Topotecan	

The subtype has been evaluated in each case with molecular diagnostic tools (FISH, Sanger sequencing, spectral karyotyping, cytogenetics)

*BM* bone marrow, *s* survival, *f* follow-up

## Conclusion

Primary alveolar rhabdomyosarcoma of the bone as a subtype of ARMS, seems to be a distinct clinico-pathological entity with challenging diagnostic difficulties and different biological behavior when compared to soft tissue ARMS. More available data might be necessary to predict not only the course of the disease, but also to develop and set up further chemotherapeutical combinations that may increase the overall survival of the patients in the future.

## Abbreviations

ALK: Anaplastic lymphoma receptor tyrosine kinase
ARMS: Alveolar rhabdomyosarcoma
CWS: Cooperative Soft Tissue Study Group
DNA: Deoxyribonucleic acid
ERMS: Embryonal rhabdomyosarcoma
FISH: Fluorescence in situ hybridization
FOXO-1: Forkhead box protein O1
HE: Hematoxylin-eosin
IHC: Immunohistochemistry
INI-1: Integrase interactor 1
LCA: Leukocyte common antigen
MRI: Magnetic resonance imaging
mTOR: Mammalian (mechanistis) target or rapamycin
Myf4: Myogenin factor 4
OS: Osteosarcoma
PAX-3/7: Paired box gene 3/7
PCR: Polymerase chain reaction
PET/CT: Positron emission tomography-computed tomography
RANKL: Receptor activator of nuclear factor kappa-B ligand
RMS: Rhabdomyosarcoma
SKY: Spectral karyotyping
TFE3: Transcription factor E3
